# Arylamine N-acetyltransferase 2 Polymorphisms and the Risk of Endometriosis

**Published:** 2018

**Authors:** Fabio Barra, Lorenzo Ferro Desideri, Carolina Scala, Simone Ferrero

**Affiliations:** Academic Unit of Obstetrics and Gynaecology IRCCS Ospedale Policlinico San Martino, University of Genoa Largo Rosanna Benzi 10, 16121 Genova, Italy

We read with interest the article by Fayez *et al* entitled “Arylamine N-acetyltransferase 2 Polymorphisms and the Risk of Endometriosis” 1 that was recently published in your journal.

These authors evaluated the association between some polymorphisms of human arylamine N-acetyltransferase 2 (NAT2) gene (in particular C481T, A803G, G857A and G590A) and the risk of endometriosis development. Specifically, it was found a significant difference in the genotype distributions and allele frequencies of NAT2 G590A polymorphisms between patients with endometriosis and healthy women; in particular, a protective role for NAT2 590A allele in the progression of endometriosis was reported. On the other hand, the authors observed a higher frequency of rapid acetylator phenotype in patients affected.

We deem that this topic appears extremely interesting, but at the same time controversial: it is known that NAT2 enzyme has a critical role in the initial biotransformation of multiple xenobiotic substances (among all aromatic amines and hydrazines) and that several polymorphisms and alleles of its gene characterize the genome of general population (with different distribution in the world) 2. Thus, it appears reasonable an eventual contribution of some NAT2 polymorphisms in the pathogenesis of endometriosis, in which the role oxidative stress (to which the systemic biotransformation of endogenous or exogenous substances may take part) has been largely demonstrated 3. On contrary, previous studies aiming to demonstrate a causative role of NAT2 polymorphisms and acetylator phenotypes in this benign hormone-dependent disease obtained controversial results 4–6.

Although the authors should be congratulated for these innovative findings, we would like to discuss an important aspect of the study. In material and methods, Fayer *et al* described that DNA was extracted from peripheral blood samples of 141 Iranian patients with surgical confirmation of endometriosis and underwent analysis by PCR-RFLP method. Anyway, authors might specify the different distribution of types of endometriosis in the enrolled population. In particular, it would be of interest to know if women had peritoneal nodules, ovarian endometriomas or deep infiltrating endometriosis (DIE) nodules, or a combination of these. On the other hand, the authors may specifically report if the risk of acetylator phenotypes for having endometriosis has been higher regardless of such endometriosis types. In fact, we think that the polymorphisms of NAT2 gene and the subsequent translated enzymes may differently contribute in these three different types of endometriosis that are known to have distinguished pathogenesis 7. Regarding this topic, it has been previously described that nodules of DIE have higher oxidative stress and inflammatory environment as well more abundant content of nerve fibers, which may cause a more aggressive clinical behavior 8. For this reason, it is conceivable that an anomalous biotransformation of biological endogenous or exogenous macromolecules may give a higher susceptibility for developing DIE.

Anyway, the results of the study by Fayez *et al* 1 are innovative and promising. Thus, in the near future, new genetic studies are awaited to better clarify the role of NAT2 in the multiple aberrant pathways that characterize endometriotic stromal cells. Additionally, it would be of interest to better understand if this protein (whenever anomalous) may also represent a suitable therapeutic molecular target. In fact, in the last year, research has been particularly active in finding new medical options to treat young women with endometriosis 9 that need a chronic treatment balancing clinical efficacy with safety 10.
